# Wildlife risk mitigation protocols reduce risk species visits and pathogen marker detection in open-air farms

**DOI:** 10.1186/s13567-025-01671-0

**Published:** 2025-11-27

**Authors:** Ángela Marín-Rojo, Gloria Herrero-García, Carmen Herranz-Benito, Patricia Barroso, David Relimpio, Teresa García-Seco, Alberto Perelló, Alberto Díez-Guerrier, Pilar Pozo, Ana Balseiro, Lucas Domínguez, Marta Pérez-Sancho, Christian Gortázar

**Affiliations:** 1 SaBio Instituto de Investigación en Recursos Cinegéticos (IREC) CSIC-UCLM-JCCM, 13071 Ciudad Real, Spain; 2 Department of Animal Health, Faculty of Veterinary Medicine, Universidad de León, 24071 León, Spain; 3 VISAVET Health Surveillance Center, Complutense University of Madrid, 28040 Madrid, Spain; 4 Department of Animal Health, Faculty of Veterinary, Complutense University of Madrid, 28040 Madrid, Spain; 5 Department of Animal Health, Mountain Livestock Institute (IGM, CSIC-ULE), Finca Marzanas, 24346 Grulleros, León, Spain; 6 MAEVA SERVET S.L. 28749, Alameda del Valle, Spain

**Keywords:** Biosafety, camera trapping, cattle, environmental nucleic acid detection, pig, small ruminants, risk mitigation, Wildlife–livestock interface

## Abstract

Outdoor farming systems may favor the dilution effect of biodiversity on pathogen exposure and contribute to biodiversity conservation through the preservation of valuable habitats. However, owing to the implicit closer interaction with wildlife, outdoor farms are also more at risk for disease maintenance at the wildlife–livestock interface. Disease control options in outdoor farming systems include the development and application of wildlife risk mitigation protocols (RMPs). However, while the nature of the proposed mitigation actions and the degree of farmer uptake have repeatedly been assessed, only limited information exists on their effectiveness. In this study, we revisited 14 farms of a pilot study to quantify the effect of applying RMPs on the detection rates of risk wildlife (assessed by means of camera traps [CTs]) and of selected pathogen markers (using sponges for environmental nucleic acid detection [ENAD]). The application of farm-specific RMPs resulted in a 30% reduction in farm visits by high-risk wildlife and an 18% reduction in the frequency of pathogen marker detection. High-risk species detection declined on 11 farms and increased on 3, all of them small ruminant farms. Regarding pathogen markers, we observed frequency reductions for four markers and increases for two markers. These changes were statistically significant for the *Salmonella* spp. marker *inv*A. At the farm level, the reduction in the detection frequency of wild boar (*Sus scrofa*) correlated with the reduction in the detection frequency of the *inv*A marker. These noninvasive methods may be relevant for assessing other interventions at the wildlife–livestock interface, regardless of the farmed species, farming system, and target pathogen.

**Supplementary Information:**

The online version contains supplementary material available at 10.1186/s13567-025-01671-0.

## Introduction

Outdoor farming systems are regarded as sustainable since they are less input-dependent and generate less waste than most indoor farming systems [1, 2]. Furthermore, some outdoor farming systems may favor the dilution effect of biodiversity on pathogen exposure risk [3, 4] and contribute to biodiversity conservation through the preservation of valuable habitats, for instance, through the creation and maintenance of lentic water bodies in arid environments [5]. However, owing to the implicit closer interaction with wildlife, outdoor farms are also more at risk for disease maintenance or emergence at the interface. This is especially relevant in the Iberian Peninsula owing to the high prevalence of endemic multihost infections shared between wildlife and livestock such as animal tuberculosis (TB) [6] and because the Peninsula represents a biodiversity hotspot in Western Europe [7]. In this context, innovating for resilience in traditional, open-air, and sustainable animal production is one way for making animal farming compatible with biodiversity conservation [4, 8].

In extensive hoofstock farming systems, disease control strategies addressing a range of risk factors simultaneously are preferable [9]. One such strategy consists in the development and application of wildlife risk mitigation protocols (RMPs) [10]. These RMPs focus mainly on avoiding or significantly reducing the direct and indirect interactions of a herd with other livestock species and wildlife. In the Iberian Peninsula, RMPs include a range of specific biosafety measures (BSMs), often targeting wildlife management (e.g., hunting) and interaction hotspots (risk points) such as water points and feeders [11, 12]. Research has evidenced that most wildlife–livestock interactions are indirect [13, 14] and take place at risk hotspots such as water points [15, 16] or feeders [17].

Once implemented, RMPs or specific BSMs should be assessed regarding their performance in terms of their efficacy in reducing wildlife–livestock interactions and improving health indicators [18, 19]. Two noninvasive methodologies are especially relevant when aiming at studying the wildlife–livestock interface and assessing the efficacy of biosecurity measures: camera trapping and environmental nucleic acid detection (ENAD). Camera trapping consists of placing automated camera traps (CTs) at strategic or random points to record the species detected on or around the farm premises. Detection rates per species can be expressed as the number of detections per CT and time [4, 6]. In turn, ENAD consists in collecting environmental nucleic acids and testing these for the presence of relevant targets such as pathogen markers [20]. Both noninvasive methodologies are rarely combined [4].

However, while the nature of BSMs proposed to farmers [11, 12] and the degree of farmer acceptance and uptake of some of these BSMs [11, 21] have repeatedly been assessed, only limited information exists on their effectiveness. For specific BSMs, such as small-scale barriers and waterhole modifications or feeder or food storage modifications, evidence has been collected on the reduction of wildlife visits [18, 22, 23] or on the improvement of herd health indicators [19]. However, quantitative information on the effectiveness of applying RMPs on the detection rates of risk wildlife and on pathogen detection remains lacking.

Our goal was to quantify the effect of applying RMPs on the detection rates of high-risk wildlife (assessed by means of CTs) and of selected pathogen markers (using ENAD). We focus on four mammals of relevance for different shared infections, namely red deer (*Cervus elaphus*), Eurasian wild boar (*Sus scrofa*), red fox (*Vulpes vulpes*), and European badger (*Meles meles*), as well as on markers of six bacterial pathogens, namely the *Mycobacterium tuberculosis* complex (MTC), *Mycobacterium avium* subsp. *paratuberculosis*, *Coxiella burnetii*, *Escherichia coli*, *Salmonella* spp., and *Brucella* spp.

## Materials and methods

Martínez-Guijosa et al. [11] developed farm-specific wildlife RMPs by which to mitigate risks at the wildlife–livestock interface in Mediterranean environments. In a recent pilot study [4], we showed that it is possible to monitor open-air farm biosafety including both hosts and pathogens. The pilot study showed that (1) short-term CT deployment generates valuable information on farm mammal communities and on the rate of farm-visits by risk species and that (2) environmental nucleic acid detection at risk points or on animals informs on pathogen marker presence in the farm environment [4, 20].

### Sampling sites and farm visits

In this study, we revisited 14 of the 15 farms included in the abovementioned pilot study. The study farms (cattle *n* = 6; small ruminant *n* = 4; and pig *n* = 4) were distributed in five regions of mainland Spain: Madrid, cattle and small ruminants; Extremadura, pig; Castilla y León, cattle; Castilla La Mancha, small ruminants; and Murcia, pig. All farms were fenced, but wildlife use of farm premises was recorded.

**Figure 1:**
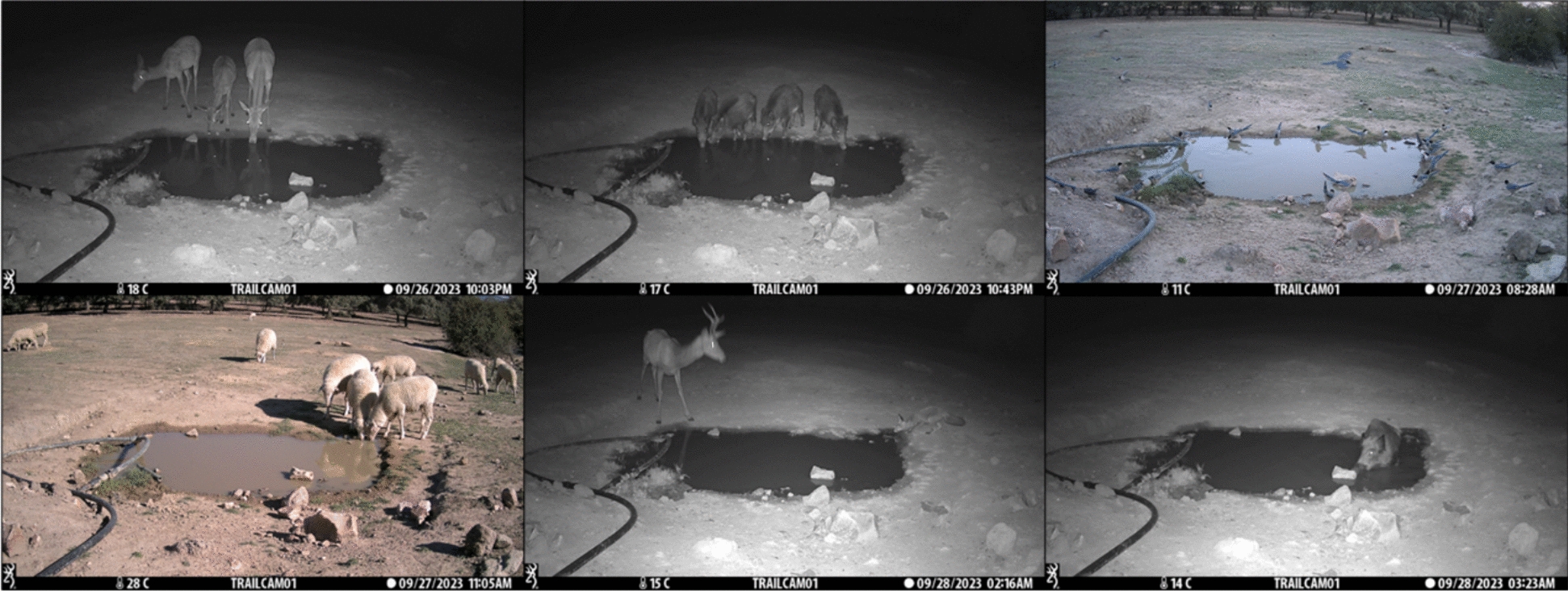
**Images captured by a camera trap at a small, unintended water point formed by a broken hose on one of the small ruminant farms.** From left to right, top to bottom (in chronological order): red deer (*Cervus elaphus*), wild boar (*Sus scrofa*), Iberian magpies (*Cyanopica cooki*), domestic sheep (*Ovis aries*), a red deer and a red fox (*Vulpes vulpes*) simultaneously, and a wild boar bathing in the water. The broken hose redirected water away from the intended trough, creating an artificial water source that attracted a variety of wild and domestic species Three of the four risk species for pathogen transmission identified in this study were recorded at this site. This situation illustrates how minor infrastructure failures can lead to indirect contact between species, highlighting the importance of implementing basic biosecurity measures to prevent such interactions.

The farms participating in this study were chosen as pilot points to run farm-specific wildlife RMPs [11]. The first round of farm visits (time 1, T1) took place in 2022 and included farmer interviews, camera trapping, and ENAD [4]. In 2022, each farmer received a detailed report of the outcome of the farm-specific wildlife RMP, including a list of recommended BSMs. The second round (T2) included new rounds of camera trapping and ENAD but no full interviews. This second sampling event took place 1 year later, in 2023, after the implementation of general and specific risk mitigation actions (Figure 1; Additional files 1 and 2). Incomplete farmer feedback on BSM uptake was gathered opportunistically at T2, and farmer compliance was not assessed.

### Camera trapping

For wildlife monitoring, Browning CTs (Browning Strike Force HD ProX, Browning Arms Company^®^, Morgan, Utah, USA) were deployed on each farm at time 1 (T1; March–June 2022) and time 2 (T2; October–December 2023). We deployed between 28 and 31 cameras per farm at each sampling time, of which 18–21 were directed toward water or food points. The remaining ten cameras were placed at random points on the farm to gain a better overview of the premises. These cameras were set to be operating for 48 h, releasing three shots when motion was detected, with a time lapse of 1 min between consecutive activations. No baits or attractants were used. By using both risk and random points, we assessed the BSM efficacy on the entire farm and not specifically at specific risk points.

This effort generated a total of 532 679 pictures, 311 309 of them collected at T1, while the rest (221 370) were obtained at T2. These pictures were screened for wildlife, discarding empty (vegetation only) pictures, and classified by species. We recorded the number of CTs detecting each relevant mammal, i.e., red deer, Eurasian wild boar, red fox, and European badger. This generated a detection frequency: number of CTs detecting a given species divided by the total number of CTs [24]. We also combined the four species as follows: number of CTs detecting red deer, wild boar, red fox, or badger divided by the total number of CTs.

### Environmental nucleic acid detection (ENAD)

ENAD sampling was performed on 20 environmental surfaces collected from risk points and on 10 animal hocks per farm at T1 (October–November 2022) and at T2 (October–November 2023). The ready-to-use GPSponge^®^ Kit (GPS genetic PCR solutions, Orihuela, Spain), prehydrated with an isotonic, surfactant, nucleic acid preservative liquid was used. For DNA extraction and purification, we used the GPSpin^®^ Microbiome Fecal DNA Kit (GPS^™^, Orihuela, Spain) according to manufacturer instructions, starting from the pellet obtained after centrifuging 900 μL of the sample for 3 min at 13 000 rpm. We tested all samples for the detection of the following pathogen markers by real-time polymerase chain reaction (PCR): *Mycobacterium tuberculosis* complex IS*6110* [25], *M. avium* subsp. *paratuberculosis* IS*900* [26]; *Brucella* sp. IS*711* [27], *Coxiella burnetii* IS*1111* [28], *Salmonella enterica* *inv*A [29], and *Escherichia coli* *uid*A [30, 31].

### Statistical analysis

To statistically analyze our results, we used Fisher’s exact statistical test, which allows us to determine whether there is a significant association between two categorical variables when the sample size is small. In this case, it would be the presence/absence of risk species or pathogen markers on each farm at T1 and T2. It was assessed using 2 × 2 contingency tables, considering a confidence level of 95%. Fisher’s exact statistical test was performed using GraphPad QuickCalcs (GraphPad software, San Diego, CA, USA). The Spearman correlation coefficient between the percentage of reduction in the detection frequency of each wildlife species and the percentage of reduction in the detection frequency of each pathogen marker was calculated using IBM SPSS Statistics (version 29.0.1, IBM Corp., Armonk, NY, USA).

## Results

**Figure 2:**
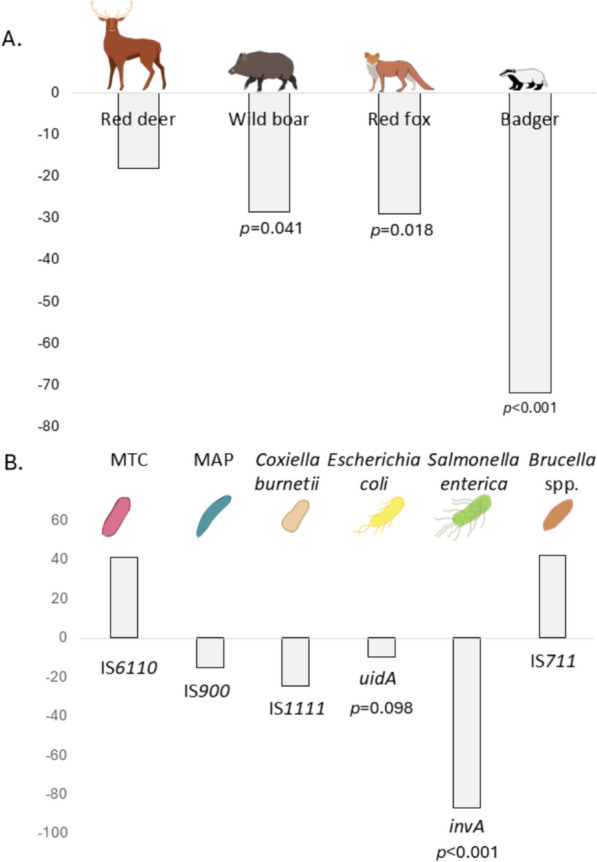
**Effects of applying risk mitigation protocols to 14 open-air hoofstock farms in Spain.** **A** Changes in the frequency (in percent) of detection of risk host species (red deer, wild boar, red fox, and badger); **B** changes in the frequency of detection of pathogen markers (IS*6110*, IS*900*, IS*1111*, *uid*A, *inv*A, and IS*711*), in % significant (*p* < 005) and marginally significant (*p* < 01) changes are indicated. MTC, *Mycobacterium tuberculosis* complex; MAP, *M. avium paratuberculosis.*

Considering the detection frequency of the four risk mammals for the set of study sites, we observed reductions in presence between 18% (red deer) and 72% (badger) in total (Table 1). The differences were statistically significant for wild boar, fox, and badger (Figure 2A). At the individual farm level, red deer appeared on 7 of 14 farms, and in 5 of them, the detection frequency decreased at T2. Wild boar appeared on 12 farms and decreased on 8 of them, including the 2 pig farms where wild boar had been detected at T2. Foxes appeared on all farms and decreased on ten of them, including all pig farms, and five of the six cattle farms. However, fox presence increased at T2 in three of four small ruminant farms. Finally, badgers were detected on ten farms, decreasing on eight of them, including five of the six cattle farms. Overall risk species detection declined on 11 farms and increased on 3, all of them small ruminant farms. This yielded an average decline of −30%, and this decline was most pronounced on cattle farms (−43%) (Table 1).

**Table 1:** **Detection frequency of four risk host species per camera trap (positive CTs of total CTs) on 14 farms before (T1) and after (T2) risk mitigation**

	CT total		Red deer	Wild boar	Red fox	Badger	Total
Farm	T1	T2	%	%	%	%	%
Cattle 1	29	28		−65	−77^*^	−65	−71
Cattle 2	30	28	−17	27	−66^**^	−100	−24
Cattle 3	30	30		−67	150	−100^$^	−46
Cattle 4	27	31	−23	34	−20	−100^*^	−41
Cattle 5	28	27		−38	−84^***^	55	−58
Cattle 6	30	29		−38	−8	−87*	−43
All cattle	174	173	−24	−18	−50^***^	−81^***^	−43
Small ru 1	29	28	−41	38	38	−100	3
Small ru 2	30	30	50	33	142^*^	50	93
Small ru 3	30	30	100	−83	450^**^	−67	54
Small ru 4	41	28	−63	−8	−63		−32
All small ru	130	116	3	−24	81^**^	−25	18
Pig 1	30	30		−100	−100^*^		−100
Pig 2	30	30	−30	−75^$^	−58^$^	−100	−55
Pig 3	27	30			−34		−34
Pig 4	30	30			−25		−25
All pig	117	120	−32	−80^*^	−48^**^	−100	−51
All farms	421	409	−18	Type="Bold">−28^*^	Type="Bold">28^*^	Type="Bold">72^***^	−30

“%” represents the change in detection frequency (in percent) between time 1 and time 2. Empty boxes indicate no detection. ^$^marginally significant Fisher’s test result; (*p* < 01), ^*^ *p* < 005, ^**^ *p* < 001, ^***^ *p* < 0001.

Small ru: small ruminants

For the six pathogen markers considered, we observed frequency reductions for four and increases for two, namely the *M. tuberculosis* complex marker IS*6110* and the *Brucella* spp. marker IS*711*. These changes were significant for the *Salmonella* spp. marker *inv*A and marginally significant for the generic *E. coli* marker *uid*A (Figure 2B). The increase in IS*6110* detection was mostly owing to new detections on two previously negative pig farms. The detection of *uid*A and *inv*A declined consistently at T2 in all three livestock species. At the farm level, we observed significant reductions for the *uid*A marker on one cattle farm and on one small ruminant farm, and for *inv*A on two cattle farms and one small ruminant farm. Detection of the *Coxiella burnetii* marker IS*1111* occurred consistently at both times in all three positive small ruminant farms, and the *Brucella* spp. marker IS*711* was detected on two small ruminant farms. The mean decline in pathogen marker detection was −18% when all markers were considered. This decline was most pronounced in small ruminant farms (−23%) (Table 2; Additional file 4). Still, at the individual farm level, we found a significant correlation between changes in the detection frequency of risk hosts and pathogen markers. Specifically, we observed a positive correlation between the percentage of reduction in the detection frequency of wild boars and the percentage of reduction in the detection frequency of the *inv*A marker (*r*_S_ = 0.68, *p* < 0.05).

**Table 2:** **Detection frequency per sponge (positive sponges of total sponges) of pathogen markers on 14 farms before (T1) and after (T2) risk mitigation**

	Sponges		IS*6110*	IS*900*	IS*1111*	*uid*A	*inv*A	IS*711*	Total %
Farm	T1	T2	%	%	%	%	%	%	%
Cattle 1	20	27	−62	100		−63^*^	−100		−54^*^
Cattle 2	20	28	185	100		53			78^*^
Cattle 3	20	30	300			−29	−100		−12
Cattle 4	19	31				−28	−80		−38^*^
Cattle 5	20	28	100	100		100	−100^***^		−6
Cattle 6	20	30		100		0	−100^*^		−23
All cattle	119	174	105	100^$^		−8	−95^***^	0	−16^$^
Small ru 1	20	30	100^$^	100	−56	−7	−67	−33	7
Small ru 2	20	30	−100	100		−67^***^	−100^*^	100	−73^***^
Small ru 3	19	30	216^$^		−79	−16	−100		−18
Small ru 4	33	27	−82^$^	−14	83	28	22		7
All small ru	92	117	−14	−29	−16	−19	−80^*^	57	−23
Pig 1	19	30				−21	−100		−25^$^
Pig 2	20	30	100			−10			0
Pig 3	20	30	−33			−2	−33		−5
Pig 4	20	30	100			21	100		12
All pig	79	120	426^$^			−3	−34		−5
All farms	290	411	41	−15	−24	−10	−85	41	−18

“%” represents the change in detection frequency (in percent) between time 1 and time 2. ^$^marginally significant Fisher’s test; (*p* < 01), ^*^ *p* < 005, ^***^ *p* < 0001.

Small ru: small ruminants

## Discussion

The application of farm-specific RMPs resulted in a 30% reduction in farm visits by risk wildlife and an 18% reduction in the frequency of pathogen marker detection. By combining short-time CT deployments with sponge-based ENAD, this innovative farm biosafety monitoring protocol enabled us to assess wildlife presence and pathogen marker detection at the same time and by noninvasive means.

The effect of the RMPs was not uniform. For instance, the detections of species regarded as high risk, i.e., the combined detection frequency of red deer, wild boar, fox, and badger, declined consistently on all six cattle farms and on all four pig farms after running the RMPs. By contrast, this indicator declined only on one small ruminant farm while it increased in the remaining three. This could suggest that cattle farmers and pig farmers were more likely to take up the mitigation actions proposed in the RMPs, possibly owing to their knowledge regarding the risk posed by endemic animal TB [6] and owing to the fear of African swine fever emergence in Europe [32], respectively. Indeed, the apparent effect of the RMPs on wild boar detection was most evident on pig farms (−80%; Table 1; Additional file 3). Comparatively, small ruminant farmers face less pressure regarding animal health and might therefore be less prone to implement time-consuming and sometimes costly mitigation actions. An alternative explanation to the low impact of RMPs on small ruminants is that implementing certain easy risk mitigation actions, such as elevating feeders and water troughs to avoid wild boars or badgers, is not as viable for small ruminants as it is for cattle given the smaller difference in body size.

The large effect of the RMPs on badger detection, especially on cattle farms (−81%) would indicate a strong effect of reducing access to feed after applying the risk mitigation actions. This coincides with previous reports from the UK [22]. However, we cannot exclude the possibility of some (undeclared) culling taking place, too. It is also interesting to note the strong effects of RMPs on fox detections, especially on cattle farms (−50%). This is relevant since foxes are potentially implicated in the cycles of several pathogens shared with livestock [33].

The effect of the RMPs on pathogen marker detection was even more variable. Markers detected on a few occasions such as IS*711* (two positive samples at T2 and one at T1) are not as relevant in terms of percentual changes as frequently detected ones. The IS*6110* marker, for instance, showed a more uniform increase from T1 to T2, notably including two pig farms. This change was not due to increases in risk species visits. However, other wildlife, including some smaller carnivores, which may be less affected by BSMs targeting larger animals (e.g., Egyptian mongoose *Herpestes ichneumon*) might also become infected and play an unknown role in MTC circulation [34]. It is important to note that only few sponge samples tested positive per farm and time, yielding only marginally significant differences. This marker had already been used in settings with known MTC circulation among cattle [20] and bison (*Bison bonasus*) [35], helping to reveal relevant risk factors. In turn, *uid*A and *inv*A, the generic *E. coli* and *Salmonella* spp. markers, were both the most detected markers and the only ones with significant reductions in frequency of detection at T2 (Table 2). These markers might constitute good general indicators of farm hygiene or farm exposure to pathogens [4]. These pathogens might also represent suitable markers of the wildlife–livestock interface. The correlation between wild boar detection and *inv*A detection suggests a link between a high-risk host (i.e., the wild boar) and a pathogen marker (i.e., *Salmonella* spp.). In this regard, the participation of the wild boar in *Salmonella* maintenance is well established [36, 37].

This study has several limitations. The first one is that we did not perform a new RMP and interview at T2, as was carried out for T1 [4]. Rather, we gathered informal farmer feedback, which generated only limited information, not allowing us to assess the degree of uptake of the mitigation actions proposed in the RMPs (Additional file 2). It is expected that most of the cheap changes to the water points and feeders were applied, while few of the expensive new fencings were implemented. Some interventions depend on third parties (neighbors, hunters, and municipal and environmental authorities) and may sometimes have been applied without consulting all relevant parties. Thus, we cannot infer whether the low impact of the RMPs in the case of small ruminant farms was a result of ineffective RMPs or poor compliance. In a similar survey focused on cattle farms, we estimated the degree of application of BSMs at one-third [11]. Another limitation is the number and timing of the CTs and ENAD. The number of CTs and the operation time was limited by the time needed by a team of two people to set up and collect the cameras while sparing time for ENAD and travel. While it is well established that longer CT deployments are needed for a full characterization of the local large-mammal community [38], we postulate that our short-term (48 h) CT deployments allowed detecting and quantifying all mammal species, which we defined as risk species. The consistent effort applied across all farms further allowed controlling bias. Other studies deployed the CTs over longer periods but used fewer cameras (e.g., two to three CTs per farm for 7–10 months on Italian poultry farms, [39]; and five CTs per farm for 3–5 months on Japanese pig farms [40]). While ENAD generally took place in the same months, CTs were generally deployed in winter/spring in 2022 and in autumn 2023. This variation could have affected wildlife abundance or farm visitation rates [14, 14]. However, we do not expect huge differences in wildlife visits since we avoided the dry summer, i.e., the main limiting season for food and water in Mediterranean habitats [5]. While our study reflects a diversity of hoofstock species and farms, it was carried out in the specific environmental context of the Iberian Peninsula. Thus, research in different geographical contexts would be desirable.

Despite all limitations, we provided the first quantitative data on the effect of implementing wildlife RMPs on the biosafety of open-air hoofstock. We found significant reductions in wildlife farm visits and in pathogen marker detection, although with variability among livestock species, wildlife species, and pathogen markers. These findings, namely that massive, short-term CT deployment in combination with sponge-based ENAD can detect changes in wildlife presence and pathogen marker detection and thus be applied to on-farm risk monitoring, can be generalized to other interventions at the wildlife–livestock interface, regardless of the farmed species, farming system, and target pathogen. We propose that combining massive, short-term CT deployments with sponge-based ENAD can be adapted to enable farmers, field veterinarians, or veterinary services to monitor farm biosafety in a broad range of settings and in a noninvasive manner.

## Supplementary Information

**Additional file 1.** **Starting date of CT deployment in the studied farms.**

**Additional file 2.** **Incomplete list of mitigation actions implemented on the studied farms.** 

**Additional file 3.** **Detection frequency of four risk host species per sponge (positive CTs of total CTs) on 14 farms before (T1) and after (T2) risk mitigation**. 

**Additional file 4.** **Detection frequency per sponge (positive sponges of total sponges) of pathogen markers on 14 farms before (T1) and after (T2) risk mitigation.**

## Data Availability

No datasets were generated or analyzed during the current study.
