# Supplementary material for: Wildlife risk mitigation protocols reduce risk species visits and pathogen marker detection in open-air farms
Source: Vet Res. 2025 Nov 27;56:237. doi: 10.1186/s13567-025-01671-0 (PMC12750572; doi:10.1186/s13567-025-01671-0)
Supplement: Supplementary file 1 — Additional file 1. Starting date of CT deployment in the studied farms. [file 13567_2025_1671_MOESM1_ESM.docx]

Additional file 1. Starting date of CT deployment in the studied farms.

| **ID** | **Region** | **Primary livestock species** | **Time 1** | **Time 2** |
| --- | --- | --- | --- | --- |
| 1 | Madrid | Cattle | 28/03/2022 | 13/11/2023 |
| 2 | Madrid | Cattle | 29/03/2022 | 15/11/2023 |
| 3 | Madrid | Cattle | 30/03/2022 | 14/11/2023 |
| 4 | Extremadura | Pig | 05/04/2022 | 23/10/2023 |
| 5 | Extremadura | Pig | 06/04/2022 | 24/10/2023 |
| 6 | Castilla y León | Cattle | 18/04/2022 | 06/11/2023 |
| 7 | Castilla y León | Cattle | 19/04/2022 | 07/11/2023 |
| 8 | Castilla y León | Cattle | 20/04/2022 | 08/11/2023 |
| 9 | Castilla la Mancha | Small ruminants | 25/04/2022 | 26/09/2023 |
| 10 | Castilla la Mancha | Small ruminants | 26/04/2022 | 25/09/2023 |
| 11 | Castilla la Mancha | Small ruminants | 27/04/2022 | 27/09/2023 |
| 12 | Murcia | Pig | 28/06/2022 | 20/11/2023 |
| 13 | Murcia | Pig | 29/06/2022 | 21/11/2023 |
| 14 | Madrid | Small ruminants | 23/05/2022 | 17/10/2023 |
