# Supplementary material for: Wildlife risk mitigation protocols reduce risk species visits and pathogen marker detection in open-air farms
Source: Vet Res. 2025 Nov 27;56:237. doi: 10.1186/s13567-025-01671-0 (PMC12750572; doi:10.1186/s13567-025-01671-0)
Supplement: Supplementary file 2 — Additional file 2. Incomplete list of mitigation actions implemented on the studied farms. [file 13567_2025_1671_MOESM2_ESM.docx]

Additional file 2. Incomplete list of mitigation actions implemented on the studied farms. C= cattle, P= pigs, SR= small ruminants.

| **Farm ID** | **Biosecurity Measures (incomplete information)** |
| --- | --- |
| 1 (C) | Increased wild boar culling efforts. Improvements in feeders and feeding practices. No changes regarding water points. Recent hunting events showed very low incidence of TB-compatible lesions in wild boars (one per ~50 individuals per hunt) and none in deer. |
| 2 (C) | Minor repairs and modifications of waterers. No other specific biosecurity measures have been implemented. |
| 3 (C) | Efforts to control water points to make them less accessible to wild boars, and supervised cattle feeding, alternating the locations for providing concentrate feed. |
| 4 (P) | Minor repairs and modifications of waterers. No other changes made. |
| 5 (P) | Minor repairs and modifications of waterers. Other biosecurity and management practices remain unchanged. |
| 6 (C) | Minor repairs and modifications of waterers. Water in several ponds has been refreshed, and this will continue periodically. Plans in the future are in place to fence off selected ponds to restrict access to wildlife only, excluding livestock (not yet implemented; awaiting approval). |
| 7 (C) | Same as ID 6: Minor repairs and modifications of waterers. Periodic water renewal in ponds already initiated. Plans to fence ponds for exclusive access by wildlife are under discussion. |
| 8 (C) | Economic constraints due to increased feed and forage prices, coupled with reduced market value of livestock due to disease status, have hindered planned biosecurity actions. |
| 9 (SR) | Minor repairs and modifications of waterers. Intense wild boar and red deer hunting, significantly reducing the population. TB-positive sheep have been culled. There are plans (future) to remove cattle entirely from the farm, maintaining only sheep. |
| 10 (SR) | Minor repairs and modifications of waterers. TB-positive sheep have been removed; intention to eliminate cattle from the farm and retain only sheep |
| 11 (SR) | Minor repairs and modifications of waterers. Fence repairs to reduce wildlife entry. Slight reduction in the cattle population. |
| 12 (P) | Minor repairs and modifications of waterers. Disinfectants are used to clean sow pens. Cleaning of pits was not feasible. Water is treated with electrolytically generated hypochlorous acid. |
| 13 (P) | Minor repairs and modifications of waterers. Disinfection practices for sow pens and use of hypochlorous acid for water treatment; pit cleaning remains impractical. |
| 14 (SR) | Minor repairs and modifications of waterers. Changes in husbandry. Previously, goats were divided into two supposedly isolated herds. However, both tested positive (including TB and paratuberculosis), prompting the owner to merge them into a single herd. |
