# Supplementary material for: Wildlife risk mitigation protocols reduce risk species visits and pathogen marker detection in open-air farms
Source: Vet Res. 2025 Nov 27;56:237. doi: 10.1186/s13567-025-01671-0 (PMC12750572; doi:10.1186/s13567-025-01671-0)
Supplement: Supplementary file 3 — Additional file 3. Detection frequency of four risk host species per sponge (positive CTs of total CTs) on 14 farms before (T1) and after (T2) risk mitigation. [file 13567_2025_1671_MOESM3_ESM.docx]

Additional file 3. Detection frequency of four risk host species per sponge (positive CTs of total CTs) on 14 farms before (T1) and after (T2) risk mitigation. % represents the change in detection frequency between Time 1 and Time 2, in percent. ^$^ marginally significant Fisher’s test result; (*p*<0.1), * *p*<0.05, ** *p*<0.01, *** *p*<0.001.

|  | **CTs total** | | **Red deer** | | | **Wild boar** | | | **Red fox** | | | **Badger** | | | **Total** | | |
| --- | --- | --- | --- | --- | --- | --- | --- | --- | --- | --- | --- | --- | --- | --- | --- | --- | --- |
| **Farm** | T1 | T2 | T1 | T2 | % | T1 | T2 | % | T1 | T2 | % | T1 | T2 | % | T1 | T2 | % |
| Cattle 1 | 29 | 28 | 0 | 0 |  | 6 | 2 | -65 | 9 | 2 | -77* | 3 | 1 | -65 | 18 | 5 | -71 |
| Cattle 2 | 30 | 28 | 13 | 10 | -17 | 16 | 19 | 27 | 16 | 5 | -66** | 3 | 0 | -100 | 48 | 34 | -24 |
| Cattle 3 | 30 | 30 | 0 | 0 |  | 6 | 2 | -67 | 2 | 5 | 150 | 5 | 0 | -100$ | 13 | 7 | -46 |
| Cattle 4 | 27 | 31 | 3 | 2 | -23 | 6 | 7 | 34 | 13 | 9 | -20 | 5 | 0 | -100* | 27 | 18 | -41 |
| Cattle 5 | 28 | 27 | 0 | 0 |  | 5 | 3 | -38 | 13 | 2 | -84*** | 2 | 3 | 55 | 20 | 8 | -58 |
| Cattle 6 | 30 | 29 | 0 | 0 |  | 5 | 3 | -38 | 9 | 8 | -8 | 8 | 1 | -87* | 22 | 12 | -43 |
| **All cattle** | 174 | 173 | 16 | 12 | -24 | 44 | 36 | -18 | 62 | 31 | -50*** | 26 | 5 | -81*** | 148 | 84 | -43 |
| Small ru 1 | 29 | 28 | 7 | 4 | -41 | 3 | 4 | 38 | 9 | 12 | 38 | 1 | 0 | -100 | 20 | 20 | 3 |
| Small ru 2 | 30 | 30 | 2 | 3 | 50 | 3 | 4 | 33 | 7 | 17 | 142* | 2 | 3 | 50 | 14 | 27 | 93 |
| Small ru 3 | 30 | 30 | 0 | 4 | 100 | 6 | 1 | -83 | 2 | 11 | 450** | 3 | 1 | -67 | 11 | 17 | 54 |
| Small ru 4 | 41 | 28 | 4 | 1 | -63 | 16 | 10 | -8 | 8 | 2 | -63 | 0 | 0 |  | 28 | 13 | -32 |
| **All small ru** | 130 | 116 | 13 | 12 | 3 | 28 | 19 | -24 | 26 | 42 | 81** | 6 | 4 | -25 | 73 | 77 | 18 |
| Pig 1 | 30 | 30 | 0 | 0 |  | 2 | 0 | -100 | 6 | 0 | -100* | 0 | 0 |  | 8 | 0 | -100 |
| Pig 2 | 30 | 30 | 10 | 7 | -30 | 8 | 2 | -75$ | 12 | 5 | -58$ | 1 | 0 | -100 | 31 | 14 | -55 |
| Pig 3 | 27 | 30 | 0 | 0 |  | 0 | 0 |  | 15 | 11 | -34 | 0 | 0 |  | 15 | 11 | -34 |
| Pig 4 | 30 | 30 | 0 | 0 |  | 0 | 0 |  | 8 | 6 | -25 | 0 | 0 |  | 8 | 6 | -25 |
| All pig | 117 | 120 | 10 | 7 | -32 | 10 | 2 | -80* | 41 | 22 | -48** | 1 | 0 | -100 | 62 | 31 | -51 |
| **All farms** | **421** | **409** | **39** | **31** | **-18** | **82** | **57** | **-28*** | **129** | **95** | **-28*** | **33** | **9** | **-72***** | **283** | **192** | **-30** |
