# Supplementary material for: Wildlife risk mitigation protocols reduce risk species visits and pathogen marker detection in open-air farms
Source: Vet Res. 2025 Nov 27;56:237. doi: 10.1186/s13567-025-01671-0 (PMC12750572; doi:10.1186/s13567-025-01671-0)
Supplement: Supplementary file 4 — Additional file 4. Detection frequency per sponge (positive sponges of total sponges) of pathogen markers on 14 farms before (T1) and after (T2) risk mitigation. [file 13567_2025_1671_MOESM4_ESM.docx]

Additional file 4. Detection frequency per sponge (positive sponges of total sponges) of pathogen markers on 14 farms before (T1) and after (T2) risk mitigation. % represents the change in detection frequency between Time 1 and Time 2, in percent. ^$^ marginally significant Fisher’s test; (*p*<0.1), * *p*<0.05, *** *p*<0.001.

|  | **Sponges** | | **IS*6110*** | | | **IS*900*** | | | **IS*1111*** | | | ***uidA*** | | | ***invA*** | | | **IS711** | | | **Total** | | |
| --- | --- | --- | --- | --- | --- | --- | --- | --- | --- | --- | --- | --- | --- | --- | --- | --- | --- | --- | --- | --- | --- | --- | --- |
| **Farm** | T1 | T2 | T1 | T2 | % | T1 | T2 | % | T1 | T2 | % | T1 | T2 | % | T1 | T2 | % | T1 | T2 | % | T1 | T2 | % |
| Cattle 1 | 20 | 27 | 2 | 1 | -62 | 0 | 2 | 100 | 0 | 0 |  | 10 | 5 | -63^*^ | 1 | 0 | -100 | 0 | 0 |  | 13 | 8 | -54^*^ |
| Cattle 2 | 20 | 28 | 1 | 4 | 185 | 0 | 1 | 100 | 0 | 0 |  | 7 | 15 | 53 | 0 | 0 |  | 0 | 0 |  | 8 | 20 | 78^*^ |
| Cattle 3 | 20 | 30 | 1 | 6 | 300 | 0 | 0 |  | 0 | 0 |  | 14 | 15 | -29 | 1 | 0 | -100 | 0 | 0 |  | 16 | 21 | -12 |
| Cattle 4 | 19 | 31 | 0 | 0 |  | 0 | 0 |  | 0 | 0 |  | 12 | 14 | -28 | 3 | 1 | -80 | 0 | 0 |  | 15 | 15 | -38^*^ |
| Cattle 5 | 20 | 28 | 0 | 1 | 100 | 0 | 2 | 100 | 0 | 0 |  | 5 | 14 | 100 | 8 | 0 | -100^***^ | 0 | 0 |  | 13 | 17 | -6 |
| Cattle 6 | 20 | 30 | 0 | 0 |  | 0 | 1 | 100 | 0 | 0 |  | 10 | 15 | 0 | 4 | 0 | -100^*^ | 0 | 0 |  | 14 | 16 | -23 |
| **All cattle** | 119 | 174 | 4 | 12 | 105 | 0 | 6 | 100^$^ | 0 | 0 |  | 58 | 78 | -8 | 13 | 1 | -95^***^ | 0 | 0 | 0 | 79 | 97 | -16^$^ |
| Small ru 1 | 20 | 30 | 0 | 6 | 100^$^ | 0 | 3 | 100 | 3 | 2 | -56 | 15 | 21 | -7 | 2 | 1 | -67 | 1 | 1 | -33 | 21 | 34 | 7 |
| Small ru 2 | 20 | 30 | 3 | 0 | -100 | 0 | 1 | 100 | 0 | 0 |  | 18 | 9 | -67^***^ | 4 | 0 | -100^*^ | 0 | 1 | 100 | 25 | 10 | -73^***^ |
| Small ru 3 | 19 | 30 | 1 | 5 | 216^$^ | 0 | 0 |  | 3 | 1 | -79 | 12 | 16 | -16 | 1 | 0 | -100 | 0 | 0 |  | 17 | 22 | -18 |
| Small ru 4 | 33 | 27 | 7 | 1 | -82^$^ | 20 | 14 | -14 | 8 | 12 | 83 | 22 | 23 | 28 | 1 | 1 | 22 | 0 | 0 |  | 58 | 51 | 7 |
| **All small ru** | 92 | 117 | 11 | 12 | -14 | 20 | 18 | -29 | 14 | 15 | -16 | 67 | 69 | -19 | 8 | 2 | -80^*^ | 1 | 2 | 57 | 121 | 117 | -23 |
| Pig 1 | 19 | 30 | 0 | 0 |  | 0 | 0 |  | 0 | 0 |  | 16 | 20 | -21 | 1 | 0 | -100 | 0 | 0 |  | 17 | 20 | -25^$^ |
| Pig 2 | 20 | 30 | 0 | 2 | 100 | 0 | 0 |  | 0 | 0 |  | 14 | 19 | -10 | 0 | 0 |  | 0 | 0 |  | 14 | 21 | 0 |
| Pig 3 | 20 | 30 | 1 | 1 | -33 | 0 | 0 |  | 0 | 0 |  | 17 | 25 | -2 | 1 | 1 | -33 | 0 | 0 |  | 19 | 27 | -5 |
| Pig 4 | 20 | 30 | 0 | 5 | 100 | 0 | 0 |  | 0 | 0 |  | 16 | 29 | 21 | 0 | 1 | 100 | 0 | 0 |  | 16 | 27 | 12 |
| All pig | 79 | 120 | 1 | 8 | 426^$^ | 0 | 0 |  | 0 | 0 |  | 63 | 93 | -3 | 2 | 2 | -34 | 0 | 0 |  | 66 | 95 | -5 |
| **All farms** | **290** | **411** | **16** | **32** | **41** | **20** | **24** | **-15** | **14** | **15** | **-24** | **188** | **240** | **-10^$^** | **23** | **5** | **-85^***^** | **1** | **2** | **41** | **266** | **309** | **-18^***^** |
